# View of the Renin-Angiotensin System in Acute Kidney Injury Induced by Renal Ischemia-Reperfusion Injury

**DOI:** 10.1155/2022/9800838

**Published:** 2022-10-22

**Authors:** Farzaneh Karimi, Maryam Maleki, Mehdi Nematbakhsh

**Affiliations:** ^1^Department of Physiology, Behbahan Faculty of Medical Sciences, Behbahan, Iran; ^2^Department of Physiology, Faculty of Medicine, Ilam University of Medical Sciences, Ilam, Iran; ^3^Water & Electrolytes Research Center, Isfahan University of Medical Sciences, Isfahan, Iran

## Abstract

Renal ischemia-reperfusion injury (RIRI) is a sequence of complicated events that is defined as a reduction of the blood supply followed by reperfusion. RIRI is the leading cause of acute kidney injury (AKI). Among the diverse mediators that take part in RIRI-induced AKI, the renin-angiotensin system (RAS) plays an important role via conventional (angiotensinogen, renin, angiotensin-converting enzyme (ACE), angiotensin (Ang) II, and Ang II type 1 receptor (AT_1_R)) and nonconventional (ACE2, Ang 1-7, Ang 1-9, AT_2_ receptor (AT_2_R), and Mas receptor (MasR)) axes. RIRI alters the balance of both axes so that RAS can affect RIRI-induced AKI. In overall, the alteration of Ang II/AT_1_R and AKI by RIRI is important to consider. This review has looked for the effects and interactions of RAS activities during RIRI conditions.

## 1. Introduction

Renal ischemia-reperfusion injury (RIRI) is a sequence of complicated events that is defined as a reduction of the blood supply followed by the recovery of perfusion and reoxygenation to the kidney [1]. The most clinical conditions of RIRI include sepsis, renal transplantation, kidney surgery, blood volume depletion, and septic shock [2, 3]. RIRI is the leading cause of acute kidney injury (AKI), which is characterized by a sudden decline in renal function, glomerular filtration rate (GFR) reduction, accumulation of nitrogen waste products, and inability to maintain fluid and electrolyte homeostasis [4–6]. Molecular mechanisms underlying RIRI-induced AKI are not fully understood, but it has been reported that several factors such as ATP depletion, reactive oxygen species (ROS) production, phospholipase activation, neutrophil infiltration, and release of vasoactive peptides contribute to the pathogenesis of renal damage [7–10]. Among the diverse mediators that take part in AKI induced by RIRI, the renin-angiotensin system (RAS) plays an important role [11–13]. Conversely, RIRI tends to alter or modulate the profile of RAS components [12, 14]. RAS components are locally found in all organs including brain, heart, adrenal glands, and vasculature components [15–17]. The kidneys are unique in this respect, and RAS components are distributed in the tubules, interstitial networks, and intracellular compartments [18].

RAS is a hormonal system with coordinated effects on the heart, vessels, and kidneys, and it is involved in regulating blood pressure and homeostasis of fluid and electrolytes [19, 20]. These important functions are performed by the main peptide of this system, angiotensin II (Ang II) [21]. There are two axes of activity for the RAS in the kidney: the conventional axis and nonconventional axis [22]. The conventional RAS include angiotensinogen (AGT), renin, Ang-converting enzyme (ACE), Ang II, and Ang II type 1 receptor (AT_1_R), and the nonconventional RAS include angiotensin 1-7 (Ang 1-7), angiotensin 1-9 (Ang 1-9), ACE2, AT_2_R, and MasR [22]. Many studies have looked for the effects and interactions of RAS activities during RIRI. It is known that RIRI alters the balance of both ACE/Ang II/AT_1_R and ACE2/Ang 1–7/MasR axes [23, 24]. Hence, the purpose of the present review is to provide an updated overview of the two axes of RAS activities in the kidney and how the effects of changes in these axes on the development and progression of AKI induced by RIRI.

## 2. The Renal View of Conventional RAS in AKI Induced by RIRI

### 2.1. Angiotensinogen and AKI Induced by RIRI

AGT is a glycosylated protein that is mainly produced by the liver, and it is secreted into plasma. However, intrarenal AGT mRNA and protein are located primarily in proximal tubular cells, and AGT can be secreted to the tubular lumen [25, 26]. AGT is a necessary component of the RAS that is broken down by renin's proteolytic activity to Ang I, which serves as the source for several active angiotensin peptides [19, 26]. Among all known biomarkers of AKI, urinary AGT (uAGT) as a biomarker of RAS overactivity is helpful for early detection of AKI [27, 28]. If renal AGT production is increased or glomerular permeability is impaired, plasma AGT can be filtered into the distal nephron and thus provide an important source of uAGT [29]. It is reported that 14 days after moderate RIRI, uAGT returns to preischemic levels followed by kidney architecture recovery while a continuous increase in uAGT was observed after severe RIRI [30]. Four weeks after RIRI, upregulation of gene encoding AGT was shown in the kidneys [31]. It is shown that uAGT levels were significantly increased in patients with AKI [32, 33]. Elevated levels of uAGT, as a powerful predictor for AKI, are associated with the severity of AKI [34]. Moreover, increased uAGT indicates that intrarenal RAS is overexpressed in patients with AKI that plays a crucial role in the development of renal injury [35, 36].

### 2.2. Renin and AKI Induced by RIRI

Renin, a protease synthesized by the juxtaglomerular apparatus of the afferent arteriole which is secreted in response to hypotension detected by baroreceptors, decreased the sodium delivered to distal nephrons and activation of the *β*1 adrenergic receptors in the sympathetic nervous system [19]. Renin cleaves AGT into Ang I, which is the initial rate-limiting step of RAS and is known as a preferred location for blockade [37]. RIRI was reported to increase the plasma release and activity of renin in the initial steps of both clinical and experimental forms of AKI [24]. Increased renin leads to increased Ang II, which induces hemodynamic changes, including afferent and efferent arteriole contraction, glomerular pressure, and GFR alteration, eventually worsening renal damage [38]. The renin response in the juxtaglomerular apparatus and collecting duct (CD) of the nephron is reduced by RIRI so that the renin content is reduced in CD in the first 8 h of reperfusion; however, after 16 h, its amount is increased. At 24 h, renin content became even more pronounced, and it increased continuously until 48 h [39]. Renin plays an important role in kidney damage due to RIRI so that inhibition of renin with aliskiren (renin inhibitor) leads to a reduction renal dysfunction and hemodynamic and histopathological alterations caused by RIRI [14, 40, 41].

### 2.3. Ang II/AT_1_R and AKI by RIRI

Ang I, made from AGT, is converted to Ang II by an Ang-converting enzyme (ACE) in lung endothelial cells [42]. In addition to the lungs, this process occurs in the vascular bed of the kidneys, heart, and brain [43]. Ang II, the main active peptide in RAS, is an octapeptide hormone with a half-life of about 30 seconds in the systemic circulation and 15 min in the cell [44]. Ang II regulates volume and fluid balance through sodium reabsorption and aldosterone secretion [45]. It also increases perfusion pressure by contracting systemic arteries and increasing systemic arterial pressure and increases cardiac contractility and stimulates the release of catecholamines from the adrenal medulla and sympathetic nerve terminals [46]. Ang II exerts its effect through two functional receptors, AT_1_R and AT_2_R. There are two subtypes of AT_1_R (AT1a and AT1b) in rodents. AT_1_R is expressed in the proximal tubule brush border, basolateral membranes and the cortical CD of the kidney, vascular smooth muscle cells of all renal vascular segments, including the afferent and efferent arterioles [16, 47]. Local RAS is involved in the progression of AKI by increasing Ang II production [22, 27].

Ischemia, or a reduction in blood flow, is the main cause of many serious disorders, including organ transplantation. According to animal studies, reperfusion of ischemic regions, particularly reoxygenation, adds to further tissue injury (reperfusion injury) [48, 49]. RIRI pathogenesis has been linked to several factors, including vascular/microvascular damage, endothelial dysfunction, rapid cell necrosis, granulocyte activation, and nitric oxide (NO)/Ang II axis regulation [49]. Pazuki-Troudi et al. have shown that RIRI (30 min of ischemia and 120 min of reperfusion) caused moderate renal injury in all animals undergoing RIRI. This impact is thought to be caused by the suppression of Ang II production. Different doses of angiotensin-converting enzyme inhibitors (ACEIs) like captopril or enalapril prevented these lesions. This implies that the RAS is implicated in the RIRI. Losartan (AT_1_R antagonist) at various dosages did not protect against RIRI lesions. These results indicate that the effects of captopril or enalapril on RIRI are not mediated by the AT_1_R, and it may be mediated by AT_2_R or mechanisms independent of Ang II. The findings of this investigation support earlier results on using ACEIs, captopril, or enalapril to prevent RIRI in rats. The ACEIs reduce bradykinin breakdown, which leads to B2 kinin receptor activation and vasodilation. This impact is thought to be caused by the generation of NO [49].

It is interesting that in other organs, such as the heart, other effects were seen in this regard. A study in alcoholic cardiomyopathy (ACM) shows that Ang II and AT_1_R contribute to the effects of alcohol on the myocardium through oxidative stress damage, the mechanism of which may be achieved by regulating nicotinamide adenine dinucleotide oxidase (NOX) [50]. Ang II increases hypercellularity, inflammation, ROS production [51], renal mesangial cell proliferation, and apoptosis and alters NO availability [52]. Numerous studies have shown increased levels of intrarenal Ang II after RIRI [22, 53, 54]. Kontogiannis and Burns demonstrated that 60 min bilateral renal ischemia followed by 24 h reperfusion caused Ang II rise in ischemic kidneys, but its level did not vary after 120 h [53]. In male Sprague-Dawley rats, unilateral renal ischemia for 60 min caused a significant increase in renal tissue levels of Ang II [54]. The increased intrarenal Ang II levels may be due to the increase in cortical renin activity, especially since the decrease of cortical ACE activity after ischemia [54, 55]. AT_1_R expression reduction was indicated in the ischemic kidney at 24 h reperfusion and recovery to sham levels at 72 h reperfusion [54].

After RIRI, the vasoconstrictor effect of Ang II decreases, possibly due to receptor reduction in vascular and tubular regions of the ischemic kidney [54]. In RIRI situation, Ang II via stimulation of the AT_1_R in the damaged kidney upregulates several proinflammatory genes and also develops tubulointerstitial fibrosis [56], but AT_1_R inhibition by losartan before RIRI reduces inflammatory factors such as interleukin 1 beta (IL-1*β*), interleukin-17 (IL-17), tumor necrosis factor-*α* (TNF-*α*) and fibrosis induced by RIRI, and as a result, tubular injury and vascular obstruction improve within 24 h of reperfusion [56–58]. Moreover, losartan reduces the progression of AKI to chronic kidney disease (CKD) by increasing renal blood flow (RBF) and stimulation of hypoxia-inducible factor 1 (HIF-*α*) [57, 58]. Upregulation of HIF as a transcription factor erythropoiesis, angiogenesis, apoptosis, and cell growth is a protective cellular response [59]. Activation of HIF1*α* (oxygen-sensitive subunit of HIF), which is primarily expressed in tubular epithelial cells, by losartan as a renoprotective mechanism can reduce renal damage in RIRI [59]. Indeed, Ang II promotes podocyte autophagy by increasing the podocyte production of autophagic genes like beclin-1 and microtubule-associated protein 1 light chain 3 (LC3) and the generation of ROS [60]. This effect interrupts the glomerular filtration barrier (GFB) and the filtration of plasma proteins and, therefore, proteinuria in RIRI that was reduced by losartan pretreatment [58]. Autophagy keeps the cellular function of renal proximal tubule cells from RIRI-induced damage and nephrotoxic drugs [61]. Autophagy precedes apoptosis in proximal tubular cells, and its deficiency exacerbates cellular injury induced by RIRI [61, 62]. Zhang et al. reported that autophagy can be renoprotective via cell survival mechanisms involving several autophagy and apoptosis-related genes in the RIRI model [63]. In the kidney, autophagy is regulated by the RAS; AT_1_R promotes autophagy in renal proximal tubular cells and increases renal tolerance to RIRI, thereby protecting the kidneys against RIR [62]. Telmisartan, an AT_1_R antagonist, has shown a renoprotective effect in RIRI; telmisartan pretreatment significantly decreased blood urea nitrogen (BUN) and serum creatinine (Cr) levels, inhibited the depletion of the antioxidant factors, and decreased lipid peroxidation induced by RIRI in the kidney [43].

Although compelling evidence indicates that biased activation of G protein-coupled receptor (GPCR) signalling, like AT_1_R signalling, plays a role in vascular homeostasis and injury, it is unclear whether clinically relevant endogenous-biased antagonism of AT_1_R signalling exists in physiological and pathophysiological circumstances. Explorations of the endogenous-biased antagonism of AT_1_R or other GPCRs may provide innovative treatment options for cardiovascular disorders [64].

### 2.4. ACE and AKI Induced by RIRI

ACE is a central component of the RAS, which converts the inactive Ang I to the active peptide Ang II and metabolizes other vasoactive peptides, such as bradykinin [65]. ACE also generates Ang 1–7 from Ang 1–9 [66]. ACE is primarily found in the endothelial cells of the lungs but also in the heart, brain, renal endothelium, and immune system cellular components [67, 68]. Like other components of the RAS, RIRI can affect the activity or expression of the ACE; the decrease of cortical ACE activity despite no change in renal expression of ACE was observed after RIRI [24, 53, 54]. The role of ACE against damage caused by RIRI was investigated using ACEIs. Injection of captopril as an ACE inhibitor in rats under 1 h RIRI and left unilateral nephrectomy inhibited Ang-II synthesis, reduced infiltration of inflammatory cells and the production of proinflammatory cytokines in the kidney, and improved renal dysfunction by decreasing Cr and BUN levels [12, 69]. Administration of captopril before ischemia or immediately after reperfusion reduced the extent of tubular necrosis and urinary casts [70]. Captopril oral gavage in rats under 60 min ischemia and 120 min reperfusion with bilateral nephrectomies reduced apoptosis, neutrophil infiltration, tissue total oxidant status (TOS), and gelatinase-associated lipocalin (NGAL) levels as a marker of renal epithelial injury [71]. Asymmetric dimethylarginine (ADMA), an endogenous inhibitor of NO synthases, decreased NO production and increased ROS production [72]. Increased generation of ADMA in plasma, which is an important risk factor for chronic kidney disease, was reduced by captopril [71]. Besides, pretreatment with zofenopril (ACEI) in rats undergoing right nephrectomy and RIR resulted in decreased protein oxidation, lipid peroxidation, and acute tubular injury, comprising nuclear condensation, brush boundary, and cytoplasmic swelling [73]. RIRI increases inducible NO synthase (iNOS) mRNA, total iNOS protein, and iNOS monomer, which are attenuated by ACEI [74]. NO production by iNOS stimulation in RIRI is harmful and has cytotoxic effect on renal tubular epithelial cells [75, 76]. Taken together, these results suggest that ACE inhibitors, including captopril, play a protective role against RIRI-induced renal damage through antioxidant, antiapoptosis, and anti-inflammatory effects.

## 3. The View of Nonconventional RAS in AKI

### 3.1. AT_2_R and AKI Induced by RIRI

AT_2_R is another functional Ang II receptor in RAS, and it is similar in molecular structure to the G protein-coupled receptor superfamily, which consists of 7 transmembrane regions [77]. The cDNA for AT_2_R encodes a protein of 363 amino acids with molecular weight of 41,220 Da; 34% of the amino acid sequence of AT_2_R is homologous with AT_1_R [78]. Although AT_2_R mRNA is not expressed in adult animals, the AT_2_R protein has been detected in fetal and newborn rat kidneys by using immunohistochemistry and Western blot analysis [79]. The renal AT_2_R was observed mainly in the vascular and tubular parts, especially the proximal tubule, collecting duct, afferent arterioles, arcuate arteries, and outer medullary descending vasa recta [67, 80]. In humans, AT_2_R mRNA was detected in the tubular organization, vessels, and glomeruli [81]. The stimulation of AT_2_R neutralizes most of the AT_1_R actions by suppressing cell proliferation and differentiation [82, 83]. It also counteracts the vasoconstrictor effects of AT_1_R via increasing the formation of kinins (bradykinin, kallikrein), NO, and guanosine cyclic 3′5′monophosphate (cGMP) and eventually dilating blood vessels and lowering blood pressure [47, 82–85]. Accordingly, the proper balance between AT_1_R and AT_2_R activation can play an important role in regulating the physiological functions of the renal and cardiovascular systems [11, 82, 86]. Studies on AT_2_R antagonists and agonists have shown a protective role for this receptor in RIRI [87, 88]. In AKI induced by RIRI, the expression of AT_2_R increased significantly [89]. After RIRI, AT_2_R blockade by PD123319 increased the RBF response to Ang II infusion in a dose-related manner in female rats [90], which might indicate the vasodilatory function of AT_2_R due to the increased NO production [91]. Infiltration of immune cells like neutrophils, monocytes, and T cells is important in RIRI and repair mechanisms [92]. RIRI causes proteinuria and triggers an inflammatory cascade mediated by infiltration of the immune cells (T cells and CD4 T cells) and proinflammatory cytokines monocyte chemoattractant protein-1 (MCP-1), IL-6, and TNF-*α* [88]. AT_2_R activation exerts anti-inflammatory effects and protects organs from injuries, including AKI [93]. Treatment with C21, as an agonist of AT_2_R, inverted these changes. Also, C21 increased CD4^+^FoxP3^+^ (regulatory T cells) and CD4^+^IL-10^+^ cells and decreased Cr, BUN, NGAL, and cell-associated kidney injury molecule-1 (KIM-1) as the markers of renal epithelial injury. These results indicate the role of AT_2_R in protecting kidneys against RIRI [88].

Lack of cytoskeletal integrity and interruption of intercellular junctions associated with ATP depletion take place in AKI [94]. The expression of AT_2_R in RIRI increased and its stimulation had beneficial renal effects. C21 pretreatment reduced renal dysfunction and preservation of tubular architecture, while PD123319 pretreatment decreased renal function in rats subjected to RIRI [84, 95]. Downregulation of proteins involved in the actin cytoskeleton regulation and the stability of epithelial intercellular connections such as Rho GTPase (the Rho family of GTPases is a family of small signalling G proteins), RhoA, and Cdc42 were reported after RIRI. Pretreatment with C21 inhibited RhoA decrease and improved cell division control protein 42 homolog (Cdc42) abundance. This data delivers evidence supporting that stimulation of AT_2_R induced renoprotective effect against AKI induced by RIRI [96]. AT_2_R activation with agonist exerts a protective effect in RIRI-related cardiohepatic dysfunction as showed by inhibited oxidative stress, downregulated inflammatory cytokines such as IL-6, IL-1, IL-17A, monocyte chemoattractant protein-1 (MCP-1), and TNF-*α*, and improved cardiohepatic depressor arm of RAS under diabetes mellitus (DM) and nondiabetes mellitus conditions [97].

### 3.2. ACE2 and AKI Induced by RIRI

ACE2, as the main component of the ACE2/Ang1-7/MasR axis, is a multifunctional enzyme that exerts its physiological effect through interaction with Ang I and Ang II peptides [98]. ACE2 catalyzes the removal of a peptide from the C-terminal end of Ang I to produce Ang1-9 that converted to Ang 1-7 by ACE [99]. ACE2 also removes phenylalanine from the C-terminal end of Ang II to generate Ang1-7 [100]. The rate of catalysis of Ang II by ACE2 enzyme is 400-fold that of Ang I; Ang II is considered the main substrate of the ACE2, so the main function of ACE2 is to transform Ang II to Ang1-7 [99, 101, 102]. Therefore, ACE2 plays a key role in the equilibrium between the two axes of vasoconstrictor (ACE/Ang II/AT1R) and vasodilator (ACE2/Ang 1-7/MasR) of RAS [103, 104]. Although renin is the limiting rate in the formation of Ang II as the primary mediator of RIRI, ACE2 through the breakdown of Ang II to Ang 1-7 plays an important role in determining the level of tissue Ang II and therefore RIRI [84, 105].

Available studies have proven a link between reducing renal mRNA expression or activity of ACE2 in RIRI [24, 106–108]. The decrease in ACE2 activity is considered a compensatory pathway for increasing Ang 1-7 production and thus a counterregulatory response to the numerous harmful effects of the increase in Ang II production [109]. The ACE2 activator, diminazene aceturate (Dize), reduced pathogenic markers and improved RIRI-induced tissue damage and renal function through upregulation of ACE2 [106, 109]. The effect of the lack of the ACE2 on RIRI in wild-type (WT) and ACE2 knockout (ACE2 KO) mice was examined, and the histologic damage scores were identical in both WT mice and ACE2 KO mice under 25 min of ischemia and 48 h of reperfusion, while in the ACE2 KO mice the levels of mRNA proinflammatory cytokines like IL-1b, IL-6, TNF-*α*, chemokines, monocyte chemoattractant protein-1 (MPC-1), apoptosis, and oxidative stress increased following RIRI [110, 111]. These data suggest a protective role for ACE2 against AKI induced by RIRI, so deletion of the ACE2 gene enhances cellular inflammation, expression of the proinflammatory cytokine, oxidative stress, and apoptosis induced by RIRI [110]. The beneficial effects of ACE2 in several models of CKD such as diabetic nephropathy, renal ablation, and unilateral ureteral obstruction also have been shown [112–114]. In addition, Ang II reduces the activity of ACE2 and Ang 1–7 through AT_1_R activation [115].

### 3.3. Ang 1-7 and AKI Induced by RIRI

Ang 1–7 is a biologically active heptapeptide found in the circulation and many tissues, such as the heart and kidney [116]. The production route for Ang 1-7 in the circulation and kidney seems different [117]. Neprilysin (NEP) is one of the major enzymes in circulation that produce Ang 1-7 from Ang I or Ang 1-9 [118]. ACE2 appears to be mainly responsible for Ang 1-7 formation in the renal tissue [119]. Its biological effects of Ang1-7 are mainly mediated through interaction with MasR and induce vasodilation, oxidative stress reduction, effects of antiproliferative and antigrowth in vascular smooth muscle cells, glomerular and proximal tubular cells, inhibit cell growth, reduce inflammation, increase urinary flow, eliminate sodium, and improve endothelial function [120–123]. Endogenous or exogenous Ang1-7 can act as a nephroprotective in several renal diseases, including AKI, hypertensive and diabetic nephropathy, glomerulonephritis, tubulointerstitial fibrosis, and preeclampsia [124]. Like other components of the RAS, the expression and amount of Ang 1-7 vary in RIRI [24]. The level of Ang1–7 in male Wistar rats, which were subjected to left nephrectomy and ischemia (45 min) followed by reperfusion (2 or 4 h) in the right kidney, was decreased [24]. The pharmacological concentrations of Ang 1-7 caused downregulation of the AT1R in cultured rat aortic vascular smooth muscle cells [125]. In addition, at 24 h after 60 min of renal ischemia, urinary concentration of Ang 1–7 increased but reached baseline levels after 72 h [54].

### 3.4. MasR and AKI Induced by RIRI

MasR, a functional receptor axis of the RAS presser, is found in multiple tissues, including the brain, heart, kidney, lung, liver, spleen, tongue, testis, and skeletal muscle [126]. In the kidney, the MasR has been localized in afferent arterioles, juxtaglomerular apparatus, proximal tubule, glomeruli, collecting ducts and the thick ascending limb of Henle [119, 124]. An increase in MasR density was observed in the glomerulus, proximal and distal tubules, and thick ascending limb of the loop of Henle after 45 minutes of ischemia and reperfusion (2 to 4 hours) in the right kidney [24]. Mas deficiency induces alterations of hemodynamics and GFR in the kidney, and Mas^−/−^ animals had reduced RBF, increased renal vascular tone, and increased GFR and urinary albumin excretion [127]. The genetic deletion of the MasR in C57Bl/6 mice induces lower urinary volume, glomerular Na^+^ accumulation, and hypertension and generates structural and molecular modifications that promote renal fibrosis [127].

RIRI in mice injected with Ang1-7 caused more significant tissue damage, which was revealed by infiltration of inflammatory cells, more severe diffuse matrix deposition, intraglomerular protein deposition, and increased cellularity [21]. The genetic deletion of MasR in C57Bl/6 mice led to glomerular hyperfiltration, which was characterized by increased inulin clearance and microalbuminuria associated with renal fibrosis and proteinuria, significant reductions in urine volume, and fractional sodium excretion [127]. RIRI (30 min of ischemia followed by 24 h of reperfusion) induced acute intense tubular vacuolization, tubular necrosis, tubular dilatation, and cast formation, and AVE0991 (MasR antagonist) improved these indexes of renal damage [128]. The genetic deletion of MasR (MasR^−/−^) did not alter the degree of renal dysfunction and inflammatory response, such as the number of systemic and renal neutrophils [128]. These results are not consistent with the study of Esteban et al. They showed that the genetic deletion of MasR leads to the absence of matrix deposition, less infiltration of inflammatory cells, reduced changes in the structure of glomeruli, and reduced inflammation [21].

## 4. Conclusion

Recent studies have identified RAS activation as an influential factor in RIRI-induced AKI. Investigation of RAS components, especially its receptors using its agonists and antagonists, has played an important role in understanding the function of this system in RIRI-induced AKI. In the conventional RAS containing Ang II/ACE/AT_1_R, the renoprotective role of ACEIs and the AT_1_R blockers in the deleterious effects of activation of this axis against ischemic AKI has been demonstrated. In the RIRI situation, Ang II via stimulation of the AT_1_R in the damaged kidney upregulates several proinflammatory genes and also develops tubulointerstitial fibrosis; so the use of losartan (AT_1_R antagonist) before RIRI improves ischemic damage by reducing inflammatory factors and apoptosis and improving renal hemodynamic response (increased RBF). In addition, ACEIs through antioxidant, antiapoptosis, and anti-inflammatory effects and also by decreasing protein oxidation, lipid peroxidation, and acute tubular injury play a protective role against RIRI-induced renal damage. Although the studies on the components of the nonconventional axis (ACE2/Ang 1-7/Ang 1-9/AT_2_R and MasR) of the RAS are less than those of the conventional axis, using activators and inhibitors has shown a protective role for the ACE2, AT_2_R, and MasR against AKI induced by RIRI. The expression of AT_2_R and MasR in RIRI is increased, which may be a compensatory mechanism for the kidney to protect itself against ischemic injury. Thus, further understanding of the role of RAS components by focusing on identifying the signalling pathways of inflammation, apoptosis, oxidant, functional impairment, and hemodynamic responses due to activation of the RAS in RIRI can play an important role in the use of agonists/antagonists or activators/inhibitors of these components against AKI caused by RIRI.

## Data Availability

No data is available.
